# Polymorphisms in *JMJD1C* are associated with pubertal onset in boys and reproductive function in men

**DOI:** 10.1038/s41598-017-17575-9

**Published:** 2017-12-08

**Authors:** Nina Mørup, Alexander Siegfried Busch, Anne Kirstine Bang, Loa Nordkap, John E. Nielsen, Ewa Rajpert-De Meyts, Anders Juul, Niels Jørgensen, Kristian Almstrup

**Affiliations:** Department of Growth and Reproduction and International Center for Research and Research Training in Endocrine Disruption of Male Reproduction and Child Health (EDMaRC), Copenhagen University Hospital, DK-2100 Copenhagen, Denmark

## Abstract

JMJD1C, a member of the Jumonji-domain containing histone demethylases protein family, has been associated with levels of sex-hormone binding globulin (SHBG) and testosterone in men, and knock-out rodent models show age-dependent infertility. The objective of this study was to investigate whether single nucleotide polymorphisms (SNPs) nearby *JMJD1C* are associated with pubertal onset in boys and with male reproduction. 671 peri-pubertal boys, 1,027 young men, 315 fertile men, and 252 infertile men were genotyped for two *JMJD1C* SNPs (rs7910927 and rs10822184). rs7910927 and rs10822184 showed high linkage. Boys with the rs7910927 TT genotype entered puberty 3.6 months earlier than their peers (p = 2.5 × 10^−2^). In young men, the number of T alleles was associated with decreased levels of SHBG, follicle-stimulating hormone (FSH), testosterone, and testosterone x luteinizing hormone, as well as increased levels of Inhibin B, Inhibin B/FSH ratio, and testis size. No significant associations with semen parameters were observed and the genotype distribution was comparable among fertile and infertile men. In conclusion, genetic variation in the vicinity of *JMJD1C* had a surprisingly large impact on the age at pubertal onset in boys as well as levels of reproductive hormones and testis size in men, emphasizing the relationship between JMJD1C and reproductive functions.

## Introduction

Male reproduction is a strictly regulated process. The process relies on proper testicular development and hormonal feedback loops that have to be well-balanced for optimal reproductive function. Numerous factors can influence the reproductive capacity of men including life style factors, endocrine disrupting chemicals and genetic and epigenetic factors^1^. Recent genome-wide association studies (GWAS) with focus on reproductive hormones, such as Sex Hormone-Binding Globulin (SHBG)^2^ and testosterone (T)^3^ in men, have revealed associations with two single nucleotide polymorphisms (SNPs) (rs7910927 and rs10822184, respectively) within or in close proximity to the jumonji domain-containing 1 C (*JMJD1C)* gene. JMJD1C is named after its Jumonji C (JmjC) domain, which is a hallmark of a large family of histone demethylases, where JMJD1C belongs to the lysine-specific demethylase 3 (KDM3) subfamily of Jumonji-domain containing proteins (reviewed in Johansson *et al*.^4^). However, the demethylase activity of JMJD1C has been disputed. Some researchers demonstrated its predicted enzymatic activity^5^ while others have been unable to reproduce this^6^. JMJD1C may possess other functions that act independently of its JmjC domain. It has been found to interact with and function as a coactivator of the androgen receptor (AR) in humans^7^ and with the thyroid hormone receptor (TR) in yeast^8^. Consequently, JMJD1C may function at several levels by regulating epigenetic patterns and by being a coactivator of principal reproductive hormone receptors.

In rodent testes, JMJD1C is mainly found in undifferentiated spermatogonia^9^. *Jmjd1c* knock-out mice exhibited age-dependent infertility, as they became infertile after 3 months of age due to progressive loss of germ cells. The same study showed that female mice deficient of *Jmjd1c* were not affected, and both serum testosterone and androstenedione levels were normal^9^. Another study used the Leydig cell line TM3 from mice to demonstrate that JMJD1C together with Steroidogenic Factor 1 bind to response elements in the promoter region of *Cyp17a1* and induce gene transcription^5^. CYP17A1 acts upstream of testosterone in the steroidogenesis pathway where it facilitates conversion of pregnenolone and progesterone to DHEA and androstenedione, respectively. In rodents, members of the KDM3 family may functionally compensate for each other in a different way from humans, although several studies have suggested important reproductive functions in humans too. Also, large GWAS have identified several SNPs in JmjC-domain containing loci associated with age at menarche in girls^10^ and type 2 diabetes in adult men and women^11^.

Here, we report an in-depth analysis of the association between two *JMJD1C* SNPs (rs7910927 and rs10822184) and male reproductive parameters in several well-characterized Danish cohorts of peri-pubertal boys, young men from the general population, and fertile and infertile men. We show that the two *JMJD1C* SNPs are not only significantly associated with circulating levels of SHBG and testosterone in accordance with previous studies, but also with age at pubertal onset in boys as well as testicular volume and levels of FSH and Inhibin B in young men.

## Results

### Genotype frequencies

The two *JMJD1C* SNPs rs7910927 and rs10822184 (Supplementary Fig. S1A) were found to be in linkage among the cohort of 671 boys from the COPENHAGEN Puberty Study (D′ = 0.97 and R^2^ = 0.91) as well as among the young men (D′: 0.99 and R^2^ = 0.90, Table 1 and Supplementary Table S1). Consequently, data from one of the SNPs is very likely to yield similar associations as the other. We did not calculate the linkage in the smaller cohorts of fertile and infertile men, since they were only genotyped for the rs7910927 SNP. The allele frequencies of the two SNPs in the cohort of young men were 0.501 for G and 0.499 for T (rs7910927) and 0.521 for C and 0.479 for T (rs10822184). The distribution of the genotypes in the adult cohorts is outlined in Table 1 together with median values of anthropometric and reproductive parameters.

**Table 1:** Cohort information.

	Young men	Fertile	Infertile
rs7910927 GG	263 (25.7)	88 (27.9)	68 (27.0)
rs7910927 TG	498 (48.7)	167 (53.0)	119 (47.2)
rs7910927 TT	262 (25.6)	60 (19.0)	65 (25.8)
rs10822184 CC	287 (28.1)	—	—
rs10822184 CT	491 (48.0)	—	—
rs10822184 TT	244 (23.9)	—	—
Median Age (Years)	19.0 (17.8–29.1)	31.7 (22.0–43.7)	34.0 (19.6–56.0)
Median BMI (kg/m^2^)	22.2 (16.0–36.5)	23.9 (16.8–45.6)	25.0 (17.0–42.0)
Median Semen Volume (mL)	3.4 (0.5–11.2)	3.6 (0.7–11.4)	3.5 (0.9–9.8)
Median Sperm Concentration (million/mL)	44.0 (0–359)	61.3 (0.9–380)	9.9(0–187)
Median Total Sperm Count (million)	141.1 (0–1377)	230.0 (5.6–1215)	34.9 (0–806)
Median Morphologically normal sperm (%)	6.5 (0–76.0)	11.0 (0–48.0)	2.5 (0–14.5)
Median Motile sperm (AB) (%)	56.3 (0–89.3)	55 (5.3–83.7)	30.0 (0–84.0)
Median Testosterone (nmol/L)	21.0 (6.3–64.1)	17.2 (7.0–35.8)	17.8 (4.1–48.2)
Median FSH (U/L)	2.7 (0.4–16.5)	3.1 (0.6–19.0)	4.8 (0.6–61.0)
Median LH (nmol/L)	3.6 (0.7–11.6)	3.2 (0.8–8.8)	3.7 (0.9–27.3)
Median Testis Size (orchidometer) (mL)	20.0 (7.0–35.0)	22.5 (7.5–37.5)	18.0 (5.0–30.0)
Median Testis Size (ultrasound) (mL)	13.3 (3.3–31.8)	14.8 (7.5–37.5)	12.0 (1.5–26.5)

For genotypes, the numbers depict the number of men with each genotype and the percentage in parentheses. For the other measures, the values are median values with lower and upper limits in parentheses.

### Association with pubertal onset

**Figure 1:**
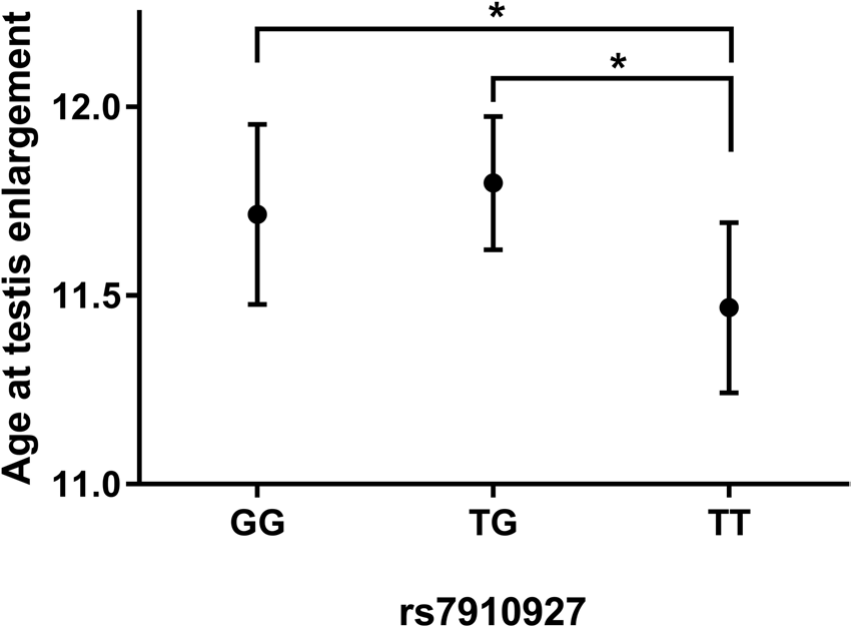
Association between pubertal onset in boys and the *JMJD1C* SNP rs7910927. Median age at testis enlargement (≥4 mL) of boys from the COPENHAGEN Puberty study stratified by rs7910927 genotype. The model is adjusted for z-BMI scores. Standard errors depict the 5–95% confidence interval. Boys with the TT genotype entered puberty 3.6 months earlier than boys with the TG or GG genotypes.

We investigated the possible effect of the *JMJD1C* SNPs rs7910927 and rs10822184 on pubertal timing assessed by testicular growth in boys from the COPENHAGEN Puberty Study. We found a significant association (p = 0.025) between age at pubertal onset and rs7910927 in a recessive model (Fig. 1) but no significant association with rs10822184. Boys with the rs7910927 TT genotype entered puberty at an age of 11.5 years, which was 3.6 months earlier than boys with the TG or GG genotypes.

### Association with reproductive parameters in young men

**Figure 2:**
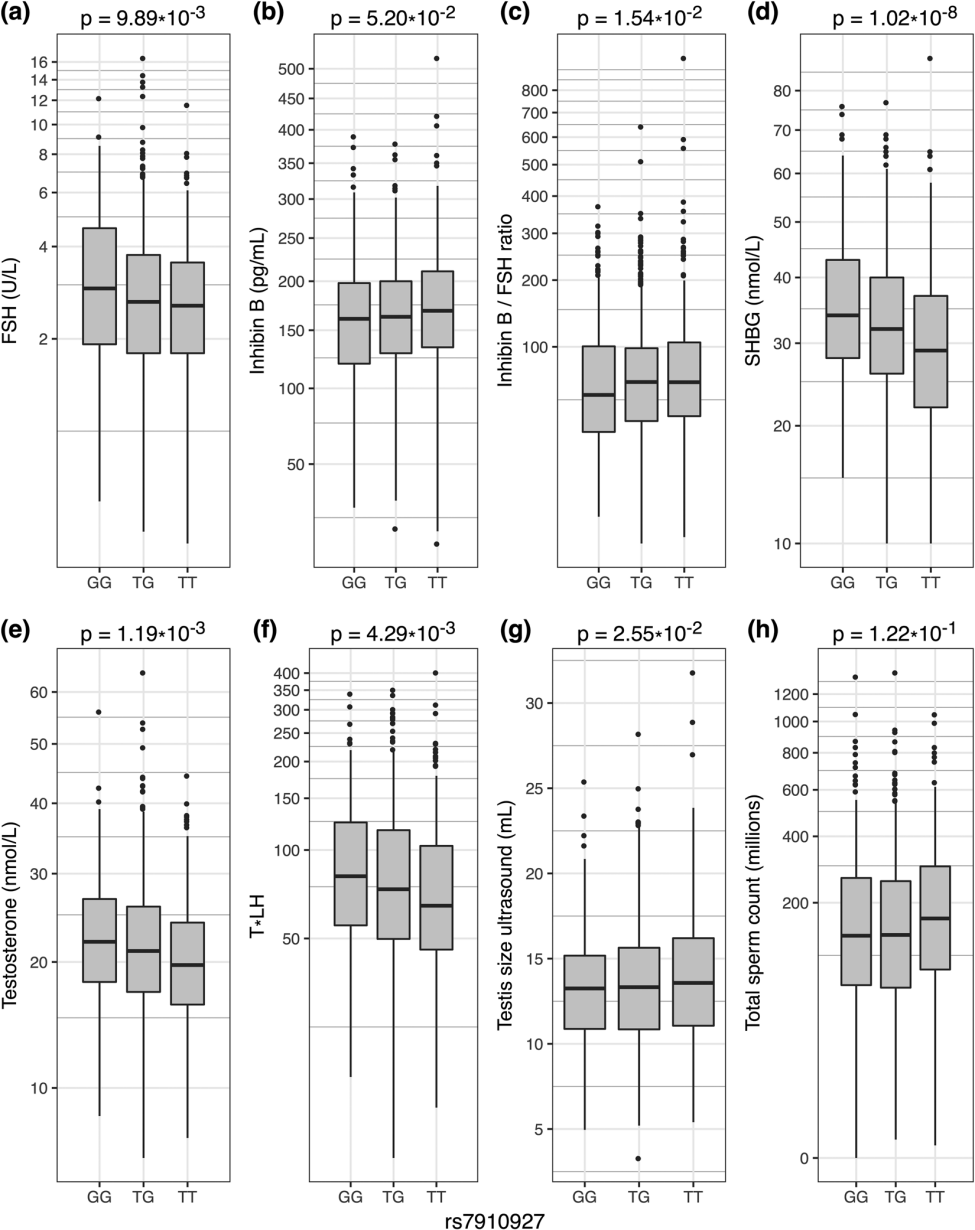
Box-plots showing reproductive hormones, testis size, and total sperm count and potential associations with the *JMJD1C* SNP rs7910927 in the cohort of young men. The models and P-values are built on additive models. Associations between JMJD1C SNP rs7910927 and (**a**) FSH, (**b**) Inhibin B, (**c**) Inhibin B/FSH ratio, (**d**) SHBG, (**e**) testosterone, (**f**) T x LH, (**g**) testis size by ultrasound, and (h) total sperm count. FSH and T x LH are presented on logarithmic scales; testosterone, SHBG, Inhibin B/FSH ratio, and total sperm count are presented on a cube root scale; and Inhibin B is presented on a square root scale in order to achieve normal distributions.

Both *JMJD1C* SNPs were found to be significantly associated with levels of SHBG (p = 1.02 × 10^−8^ (rs7910927); p = 2.0 × 10^−7^ (rs10822184), Fig. 2d, Table 2, and Supplementary Fig. S2) and total T (p = 1.19 × 10^−3^ (rs7910927) and p = 5.4 × 10^−4^ (rs10822184), Fig. 2e, Table 2, and Supplementary Fig. S2) in young men from the general Danish population. The levels of SHBG and T decreased with increasing numbers of the minor alleles (T) of both variants. In addition, both SNPs were found to be significantly associated with levels of FSH (p = 9.89 × 10^−3^ (rs7910927) and p = 1.5 × 10^−3^ (rs10822184), Fig. 2a, Table 2, and Supplementary Fig. S2) with decreasing levels of FSH as a function of increasing number of minor alleles (T). Both SNPs were also significantly associated with levels of Inhibin B (p = 5.2 × 10^−2^ (rs7910927) (not significant after log-transformation) and p = 2.0 × 10^−2^ (rs10822184), Fig. 2b, Table 2, and Supplementary Figure S2), with increasing inhibin B levels as a function of increasing number of minor alleles. No significant associations were observed with Luteinizing hormone (LH) or Estradiol levels (Table 2). In summary, men with the TT genotype (for both SNPs) had significantly lower T, SHBG and FSH levels but higher Inhibin B levels (only significant for rs10822184) (Table 2).

**Table 2:** *JMJD1C* SNPs and reproductive parameters in young men.

	rs7910927					rs10822184				
	N	GG	TG	TT	P-value	N	CC	CT	TT	P-value
		Mean	Mean	Mean			Mean	Mean	Mean	
FSH (U/L)	998	2.88	2.63	2.49	**9.89e-03**	996	2.88	2.63	2.46	**5.29e-03**
Inhibin B (pg/mL)	995	156.81	161.33	169.50	5.20e-02	993	155.54	162.23	169.95	**2.77e-02**
InhibinB/FSH ratio	995	59.29	66.14	72.84	**1.54e-02**	993	58.89	66.46	73.94	**6.61e-03**
LH (U/L)	998	3.68	3.60	3.45	1.98e-01	996	3.71	3.59	3.43	1.02e-01
Testosterone (nmol/L)	993	22.19	21.46	20.08	**1.19e-03**	991	22.18	21.52	19.88	**2.29e-04**
Testosterone/LH ratio	993	6.07	6.08	5.90	5.99e-01	991	6.02	6.11	5.88	4.99e-01
Testosterone x LH	993	81.04	75.90	68.14	**4.29e-03**	991	81.57	75.99	66.93	**7.55e-04**
cFT (pmol/L)	991	470.71	472.79	461.50	6.42e-01	989	472.43	475.03	456.10	2.88e-01
cFT/LH ratio	991	136.28	144.82	144.26	3.45e-01	989	137.05	145.37	143.05	3.93e-01
Estradiol (pmol/L)	997	87.07	86.15	83.93	4.59e-01	995	87.64	85.51	84.21	3.96e-01
Testosterone/Estradiol ratio	993	273.36	281.35	263.2	3.20e-01	991	271.17	284.79	260.33	1.26e-01
cFT/Estradiol ratio	991	5.81	6.19	6.01	3.35e-01	989	5.79	6.27	5.94	1.46e-01
SHBG (nmol/L)	996	34.75	32.22	28.99	**1.02e-08**	994	34.54	32.1	29.01	**2.58e-08**
Semen volume (mL)	964	3.43	3.28	3.36	3.15e-01	964	3.45	3.25	3.38	1.01e-01
Sperm concentration (millions)	967	41.31	42.36	46.89	2.86e-01	967	41.31	42.73	46.73	3.84e-01
Total sperm count (millions)	963	139.88	134.93	156.68	1.22e-01	963	141.01	135.01	156.50	1.46e-01
Morphologically normal sperm (%)	953	6.85	7.19	7.42	4.29e-01	953	6.92	7.23	7.45	4.65e-01
Motile sperm (AB) (%)	934	56.22	54.49	53.87	2.66e-01	933	56.47	54.31	53.95	1.53e-01
Testis size (orchidometer) (mL)	999	19.94	20.41	21.03	**3.25e-02**	997	20.05	20.34	21.05	**4.71e-02**
Testis size (ultrasound) (mL)	995	13.15	13.58	14.05	**2.55e-02**	994	13.20	13.54	14.09	**2.44e-02**

Associations between rs7910927 and rs10822184 and male reproductive parameters in young men assuming additive genetic models. N: number of men. Values are geometric mean values. P-values below 0.05 are considered significant and are marked in bold.

The ratio of Inhibin B and FSH as well as the ratio of T and LH reflects the efficiency of the feedback loop between the gonads and the pituitary gland. We found significant associations between the two SNPs and the Inhibin B/FSH ratio (p = 1.54 × 10^−2^ (rs7910927) and p = 1.9 × 10^−2^ (rs10822184), Fig. 2c, Table 2, and Supplementary Fig. S2), reflecting an association with Sertoli cell function. Men with the TT genotype (of both SNPs) had a higher Inhibin B/FSH ratio indicating a better Sertoli cell function or ability to respond to FSH stimuli. No significant differences were found for the T/LH ratio (reflecting Leydig cell function) or the T/Estradiol ratio (a proxy for aromatase activity) (Table 2). However, both *JMJD1C* SNPs were significantly associated with T x LH (p = 4.3 × 10^−3^ (rs7910927) and p = 7.5 × 10^−4^ (rs10822184), Fig. 2f, Table 2, and Supplementary Fig. S2), where men with the TT genotype had lower T x LH compared to men with the other genotypes (for both *JMJD1C* SNPs).

We did not find any significant associations of semen parameters with the *JMJD1C* SNPs, although there was a tendency that men with the TT genotype had higher sperm concentration and higher total sperm count compared to men with other genotypes (for both *JMJD1C* SNPs, Fig. 2h, Table 2, and Supplementary Fig. S2). However, we detected a significant association between the *JMJD1C* SNPs and testicular volume (p-values for testis volume measured by ultrasound: 2.55 × 10^−2^ (rs7910927) and 2.4 × 10^−2^ (rs10822184)) (Fig. 2g, Table 2, and Supplementary Fig. S2) where the testis size increased with increasing numbers of minor alleles (T). Men with the TT genotype of both *JMJD1C* SNPs had significantly larger testes (approximately 1 ml) (Table 2).

### Genotype frequencies among fertile and infertile men and associations with reproductive parameters

We investigated the frequency of one of the *JMJD1C* SNPs (rs7910927) in two other adult cohorts representing infertile (n = 252) and fertile men (n = 315). The distribution of genotypes is listed in Table 1. A chi-squared test revealed no significant differences in genotype frequencies when comparing each of the groups to the young men representing the general population. Moreover, no significant differences were found when fertile men were compared to infertile men. Among the fertile men, but not the infertile men, we found a significant association between the rs7910927 SNP and estradiol levels (see Supplementary Table S2 and S3). Similar, but non-significant, effects on reproductive hormone levels as observed with the young men, was observed among the fertile men (Table 2 and Supplementary Table S2). However, the tendencies differed among the infertile men where men with the TG genotype (rs7910927) had highest FSH, lowest Inhibin B/FSH ratio and lowest T (Supplementary Table S3).

### Expression of JMJD1C and AR in tissues with different genotypes

In order to investigate the possible effect of the *JMJD1C* SNPs on JMJD1C and AR protein expression we performed immunohistochemical staining of testicular biopsies from men with different *JMJD1C* rs7910927 genotypes (6 TT, 6 TG and 5 GG). We observed that JMJD1C was primarily expressed in the nucleus of Sertoli and Leydig cells as well as germ cells until the round spermatid stage (see Supplementary Fig. S3). There was a slight tendency towards stronger staining of JMJD1C in biopsies originating from men with the TT genotype (see Supplementary Fig. S3). However, due to the nature of immunohistochemistry (IHC), as a semi-quantitative method, along with differences in fixation times of the biopsies, these results were not directly quantitatively comparable. No apparent differences in AR expression were observed between genotypes in the IHC experiments (evaluated by microscopy) and in all cases the AR protein was confined to the nuclei of Sertoli, Leydig and peritubular cells (see Supplementary Fig. S3).

## Discussion

In this study, we demonstrated that genetic variants related to *JMJD1C* were significantly associated with onset of puberty in boys, with a range of reproductive hormones (T, SHBG, FSH and Inhibin B) as well as testis size in adult men.

It is surprising that out of the large family of histone demethylases, JMJD1C appears to be modulating several reproductive functions both in adolescence and adult life. Besides the associations identified in our study, other studies have demonstrated associations of *JMJD1C* SNPs with reduced testicular function^9^, reproductive hormones^2,3^, puberty^10^ as well as intracranial germ cell tumours^12^. No other histone demethylase shows the same number of associations with reproductive functions. Albeit a significant association with untransformed inhibin B levels was observed, the significance was lost after log-transformation for rs7910927. This could be due to differences in the distribution of inhibin B levels in men with different genotypes. The special link to reproductive function is, however, evident and may be due to the distinct feature of JMJD1C, which, besides its histone demethylase activity, most likely can interact directly with both the AR and the TR. Consequently, the impact of genetic variants in *JMJD1C* may have different effects in foetal and adult life or in different cell types depending on the exact location of the variants inside the *JMJD1C* gene (reviewed in Johansson *et al*.^4^). We speculate, that in cases where JMJD1C is engaged in histone demethylation, as described in rodent knockout studies^9^, it can be affected by genetic variants found in the JmjC-domain responsible for this function, but is not affected by variants in the AR or TR binding-domains. However, in cell types or developmental windows where the AR or TR is expressed, JMJD1C may also be engaged in co-activation of these receptors. In such cases, genetic variants in AR and TR binding-domains may influence the receptor activation and not the histone demethylase activity. Despite that the variants investigated in our study, are found outside the coding region of *JMJD1C*, they might affect *JMJD1C* promoters or enhancers only effective under certain conditions. However, with the potential co-activation of both the AR and TR, it is likely that the effects on reproductive hormones are mediated through an effect on Sertoli cells. We found that JMJD1C was expressed in the testis and primarily confined to the nucleus of Sertoli and Leydig cells as well as germ cells until the round spermatid-stage. Moreover, the highly significant association with SHBG might be different from direct effects on the hypothalamic-pituitary-gonadal axis (HPG-axis), as SHBG is not produced in the testes or brain but in the liver. Future studies are needed to dissect the possible functional roles of JMJD1C in reproductive tissues.

The TT genotypes of both *JMJD1C* SNPs seem to be associated with the most optimal testicular function. The carriers of this genotype have lower FSH but higher Inhibin B levels, indicating that their Sertoli cells respond better to FSH and do not need as much stimulation to support their function. Furthermore, since the men have lower T and SHBG (and a tendency to lower LH) we speculate that this could be due to their Sertoli cells being more sensitive to T stimulation, producing higher amounts of inhibin B (significant for rs10822184) and consequently supporting spermatogenesis better. In support of this, men with the TT genotype had lower T x LH index than the other men, and this was not affected by BMI. Men with the TT genotype also had larger testes (significant for both SNPs) and a non-significant tendency towards higher total sperm count. Moreover, murine *Jmjd1c*-knockout models have shown an age-related decrease in spermatogenesis, which could indicate sub-optimal Sertoli cell function leading to a gradual loss of germ cells^9^. We speculate therefore that JMJD1C may play a role in the development of human Sertoli cells.

In line with this hypothesis, we observed that boys with the TT genotype entered puberty 3.6 months earlier. A previous study found that self-reported onset of puberty was connected to subsequent semen quality and reproductive hormones in healthy young men^13^. The study showed that men entering puberty earlier and later than their peers had poorer semen quality and smaller testes. Furthermore, their hormone profiles also differed compared to the men that entered puberty at the average time. We do not have longitudinal data on reproductive parameters from the boys from the COPENHAGEN puberty study and the *JMJD1C* genotype did not change pubertal onset enough for the boys to be categorized as entering early or late. We nevertheless believe that there might be a connection between the earlier pubertal onset in the boys with the TT genotype and the potential better testicular function observed among young men with the TT genotype.

## Conclusion

Genetic variations near the histone demethylase *JMJD1C* locus significantly affect pubertal onset in boys as well as circulating levels of several reproductive hormones and testis size in adult men. How JMJD1C functionally mediates these effects remains to be elucidated.

## Methods

### Study populations

We included 671 boys and adolescents from the COPENHAGEN puberty study, 1,027 young Danish men representative of the general population, 315 fertile men, and 252 infertile men.

### The COPENHAGEN puberty study

Participants were recruited as part of two population-based cohort studies of healthy Danish children and adolescents. Detailed information about the study has been published previously^14^. The COPENHAGEN Puberty Study^14,15^ (ClinicalTrials.gov ID: NCT01411527) is a cross-sectional study that has been conducted at ten schools in the Copenhagen area between 2006–2014. A total of 3,101 boys were invited to join the study, of which 767 boys were examined (overall participation rate, 25%). The boys with DNA available (n = 671) were included in the present study. Physical examinations of the boys included pubertal staging of genital development according to Tanner’s classification and testicular volume^16^. Testicular volume of 4 mL and above (at least one testis) was considered to be a marker of pubertal onset.

### Young men from the general population

Young Danish men 18–25 years of age were invited to participate in a study of testicular function. The men answered a comprehensive questionnaire, underwent a physical examination, delivered a semen sample, and had a blood sample drawn. More information about the cohort and a description of exclusion criteria can be seen in Supplementary Materials and Methods and Supplementary Fig. S1B. The young men represent the general population and were recruited without any prior knowledge about their fertility status and thereby they represent both fertile and infertile men.

### Fertile men

The group of fertile men comprised two groups examined at the Department of Growth and Reproduction; one examined in 1996–1998, which has been described previously^17^ and the other investigated in 2012–2014 following the same protocol. For both study periods, the men were invited to participate in the study when their female partners were examined routinely during the second trimester of their pregnancy. The men were invited to the study if they were born and raised in Denmark, resided in the local referral area, were 20–45 years of age, and the current pregnancy was achieved by natural conception (i.e. not the result of fertility treatment). They underwent an examination program similar to that described for the men from the general population including a similar questionnaire. Exclusion criteria are described in Supplementary Materials and Methods and Supplementary Fig. S1B.

### Infertile men

The group of infertile patients consisted of 405 infertile men who had been examined for male infertility in the out-patient clinic at the Department of Growth and Reproduction, Copenhagen University Hospital, Denmark, in 2013 and 2014. During their work-up, the men delivered one or two semen samples for analysis and they had a blood sample drawn at the first visit. For more information and a description of exclusion criteria see Supplementary Materials and Methods and Supplementary Fig. S1.

### Semen samples

All adult men provided a semen sample by masturbation in a room close to the semen laboratory. The semen analysis is thoroughly described in Jørgensen *et al*.^18^. Analyses performed included volume measurement and assessment of sperm concentration, total sperm count, motile sperm cells, and morphologically normal sperm cells. Furthermore, the abstinence time was noted.

### Reproductive hormone profiles

Blood samples were drawn between 0800 h and 1400 h. Hormone measurements of the infertile men were performed immediately following blood sampling. Blood samples from the peri-pubertal boys, the young men and fertile men were centrifuged and serum frozen at −20 °C until analysed in batches.

Serum levels of FSH, LH, SHBG, T, estradiol, and Inhibin B were determined by immuno-based techniques as described in^19^. Free T (cFT) was calculated on the basis of the measured serum concentrations of total T and SHBG using the method of Vermeulen *et al*.^20^ and a fixed albumin concentration of 43.8 g/L.

Reproductive hormones (the same as mentioned above) in blood samples from boys from the COPENHAGEN puberty study were analysed with similar methods as described by Busch *et al*.^21^.

### Genotyping

EDTA-preserved peripheral blood was used for isolation of genomic DNA using SEV AS1010 DNA purification kits on a Maxwell 16-MDx instrument (Promega, Madison, WI, USA). The DNA concentration was quantified on a NanoDrop ND-1000 spectrophotometer (Saveen Werner, Limhamn, Sweden). The DNA samples were analysed using the KASP^TM^ SNP genotyping assays specific for the two *JMJD1C* SNPs rs7910927 and rs10822184. A thorough description of the method can be found in Supplementary Materials and Methods.

### Immunohistochemistry

Seventeen testis tissue samples from the archives of Department of Growth and Reproduction were used for immunohistochemistry (IHC) with antibodies against JMJD1C and the androgen receptor. These samples were biopsies taken from the contralateral testicle from patients orchiectomised because of germ cell neoplasia *in situ* (GCNIS) or invasive testis cancer. Only biopsies, without GCNIS or any other pathology were selected for the study, and all specimens had normal histology and tubules with complete spermatogenesis. The biopsies were obtained from the surgery departments at different times of the day, so the tissue fixation time varied, which could influence the quality of the tissue. Moreover, DNA originating from blood samples of the same patients was available and genotyping for the *JMJD1C* SNP rs7910927 revealed 6 TT, 6 TG and 5 GG specimens. IHC was performed as previously described^22^. Antigen retrieval was conducted with a pressure cooker (Biocare medical decloaking chamber, Concord, CA, USA) in TEG buffer (10 mM Tris, pH 8.5). The JMJD1C monoclonal mouse anti-human antibody (WHO221037M3, Sigma Aldrich, Saint Louis, MO, USA) was used in dilution 1:2,500 (0.2 μg/ml) and the monoclonal mouse anti-human androgen receptor antibody (MS-443-p, ThermoFisher Scientific, MI, USA) was diluted 1:100 (2 μg/ml). Peroxidase reaction development was performed with diaminobenzidine which resulted in a brown colour.

### Data processing and statistical analyses

Hardy-Weinberg equilibrium as well as associations between single SNPs and reproductive parameters were analysed by use of the R package ‘SNPassoc’^23^. All analyses were built on additive (codominant) genetic models unless otherwise stated. Linkage disequilibrium was calculated by use of the R package ‘Genetics’^24^.

Statistical analyses of pubertal timing (COPENHAGEN puberty study) was performed by use of the statistical software SAS (Probit analysis: proc lifereg) version 9.4 (SAS Institute, Inc). Detailed information about the proc lifereg procedure has been given previously^21^. In the analysis, age at pubertal onset was adjusted for BMI z-score WHO age-specific body mass index scores^25^. If longitudinal data was available, a mean of all individual BMI z-scores was calculated.

The T/LH and Inhibin B/FSH ratios as well as T x LH were calculated. Most of the data was transformed to achieve normal distribution: Inhibin B/FSH ratio, T, T/LH, cFT, SHBG, semen volume, sperm concentration, and total sperm count by cubic root transformation; FSH, LH, and T x LH by log transformation, and Inhibin B and estradiol by square root transformation.

The following confounders were included in the analyses; FSH, LH, Inhibin B, and SHBG were adjusted for BMI and time of blood sampling; T and estradiol were adjusted for BMI, time of blood sampling and smoking. Semen parameters (volume, concentration, total sperm count and morphology) for the young men were adjusted for abstinence time (divided into three variables – below 24 h, 24–96 h and above 96 h), fever, cryptorchidism, presence of varicocele, previous infections in the epididymis, smoking, hash smoking (except in the fertile cohort), and maternal smoking during pregnancy. Sperm motility was, in addition to the above-mentioned confounders, also adjusted for the time from sample delivery to semen analysis. For the infertile men, semen parameters were adjusted for the same parameters as for the young men except from maternal smoking during pregnancy due to lack of information about this.

### Ethical Approval

The study was approved by the Ethical Committee of the Capital Region of Denmark (H-KF-289428, H-KF-282214, H-4-2010-138, 2012-41-1390, H-16019637). The study was conducted in accordance with the Helsinki II Declaration. All men received written information and informed consent was obtained from all participants. All boys from the COPENHAGEN Puberty Study and their parents received written information and informed consent was obtained.

### Data availability

The datasets analysed during the current study are not publicly available due to Danish ethical rules on human pseudo anonymous data protecting the privacy of cohort participants. To meet requirements of reproducibility we provide two files (Supplementary Files 1 and 2) with genotypes and their link to single endpoints. The order of the genotypes (individuals) has been scrambled between each endpoint.

## Electronic supplementary material

Supplementary information

Supplementary file 1

Supplementary file 2
