# Supplementary material for: Polymorphisms in JMJD1C are associated with pubertal onset in boys and reproductive function in men
Source: Sci Rep. 2017 Dec 8;7:17242. doi: 10.1038/s41598-017-17575-9 (PMC5722903; doi:10.1038/s41598-017-17575-9)

Polymorphisms in *JMJD1C* are associated with pubertal onset in boys and reproductive function in men.

Nina Mørup, Alexander Siegfried Busch, Anne Kirstine Bang, Loa Nordkap, John E. Nielsen, Ewa Rajpert-De Meyts, Anders Juul, Niels Jørgensen, and Kristian Almstrup

Supplementary Methods:

Young men from the general population

The setup for this cohort has been extensively described in previous publications [18,19,26]. Since 1996, an ongoing study on testicular function of young Danish men from the general population has been conducted at the Department of Growth and Reproduction, Copenhagen University Hospital, Denmark. The invited men are 18-25 years of age and unselected regarding fertility or semen quality status. These men are included during a compulsory medical examination before being considered for military service and are invited irrespectively of whether they are considered fit for military service or not, and are therefore considered representative of the general population of young Danish men.

On the day of study participation, they handed in a comprehensive questionnaire on lifestyle and medical history including information on previous and/or current systemic and genital diseases, lifestyle factors, medication, cigarette smoking status, and information regarding fever above 38°C (100.4°F) within the previous 3 months. All participants underwent a physical examination, including measurement of body weight and height as well as ultrasound examination of the scrotum as a measure of testicular volume. Additional evaluation of testicular volume by use of a Prader orchidometer and the possible presence of a varicocele (stage 1–3) or hydrocele was also recorded. A semen sample was obtained by masturbation and a blood sample for analysis of reproductive

hormones and isolation of DNA. Prior to participation they had been recommended to abstain from ejaculation for at least 48 hours. Participants received a financial compensation for their attendance (500DKK, approximately 65€).

Of the 1,077 men examined in the period of 2010-2013, 1,027 men included in the study and genotyped for *JMJD1C* SNPs rs7910927 and rs10822184. 50 men were excluded (Supplementary Fig. 1) due to missing DNA sample (n = 26), use of anabolic steroids (n = 13), serious scrotal or testicular injury (n = 1), serious illness or medication (n = 9), or obstructive azoospermia (n = 1). Thus, data from 1,027 men were included. A few men had missing values of some parameters, including e.g. age (n = 5), BMI (n = 5), and abstinence time (n = 2). These men were excluded from analyses where the values were included as confounders.

Fertile men

DNA samples were available for 323 of the fertile men. Eight men were excluded due to; use of anabolic steroids (n = 1), serious scrotal or testicular injury (n = 1), orchidectomy (n = 1), and serious illness or medication (n = 5) (Supplementary Fig. 1). Thus, in total 315 fertile men were included and genotyped for the *JMJD1C* rs7910927 SNP.

Infertile men

For this study, data from the first semen sample was used in the analyses unless the abstinence time for the second semen sample was more optimal (between 48 and 96 hours). At the day of attendance in the out-patient clinic, their medical history was obtained by standardized questioning including lifestyle and medical history. A physical examination was performed, including ultrasound examination of the scrotum.

Of the 405 infertile men, 153 men were excluded due to; use of anabolic steroids (n = 7), serious scrotal or testicular injury (n = 11), obstructive azoospermia (n = 11), missing lifestyle data (n = 16), cilia dyskinesia (n = 2), chromosomal abnormalities (n = 38), orchidectomy (n = 14), serious illness or medication (n = 43), antibodies in semen or recent infection (n = 3), and tumour/GCNIS detected during work-up (n = 8) (Supplementary Fig. 1). Thus, data on 252 infertile men were included and genotyped for the *JMJD1C* rs7910927 SNP. Men with minor infertility causes including cryptorchidism and varicocele were included in the study, but adjusted for in the analyses.

Genotyping:

For genotyping of the *JMJD1C* SNPs, KASP^{TM} SNP genotyping assays were used, which facilitate bi-allelic discrimination through competitive PCR and incorporation of a fluorescent resonance energy transfer quencher cassette. Genotyping of the young Danish men was performed at LGC Genomics (Hoddesdon, UK) whereas all other samples were analysed at Copenhagen University Hospital. KASP^{TM} genotyping assays were designed towards the following sequences: rs7910927 AACTAAAATAGAAAA[K]TTATATGCATTAAAA and rs10822184 CYCCTYAAGCAGA[Y]GTACARCCATCCT. Quantitative assessment of the competitive PCR reaction was performed on a Stratagene Mx3000P qPCR instrument (Agilent Technologies, Santa Clara, USA) or on a QuantStudio5 (ThermoFisher Scientific) with the following program: 94°C for 15min, 10 cycles of 94°C for 20s and 61-55°C (drop 0.6°C each cycle) for 60s, 30-35 cycles of 94°C for 10s and 55°C for 60s, finish at 25°C for post-read. 5-20 μg DNA was used and only samples that clearly clustered into distinct genotypes were considered. rs7910927 was analysed in all cohorts whereas rs10822184 was only analysed in the boys and young men cohorts. Allele distributions were consistent with the Hardy-Weinberg equilibrium (p-value for rs7910927: 0.4162 and p-value for rs10822184: 0.2340). rs7910927 is an intronic SNP located inside the *JMJD1C* gene at chromosome 10 in position

63379150 (GRCh38.p7) on the forward strand. rs10822184 is located upstream of the *JMJD1C* gene at chromosome 10 in position 63577393 (GRCh38.p7) on the forward strand.

Supplementary table and figure legends:

Supplementary Table S1: *JMJD1C* genotype frequencies in the young men combined. The two SNPs are highly linked. The Linkage Disequilibrium is: D': 0.99 and $R^2 = 0.90$.

Supplementary Table S2: Associations between rs7910927 and reproductive hormones etc. in fertile men. N: number of men. Values are geometric mean values. P-values below 0.05 are considered significant and are marked in bold.

Supplementary Table S3: Associations between rs7910927 and reproductive hormones etc. in infertile men. N: number of men. Values are geometric mean values.

Supplementary Figure S1: Overview of the JMJD1C locus and the approximate position of the two investigated SNPs (a) and a flowchart showing the exclusion criteria for the adult male cohorts (b).

Supplementary Figure S2: Box-plots showing reproductive hormones, testis size, and total sperm count and potential associations with the *JMJD1C* SNP rs10822184 in the cohort of young men. The models and P-values are built on additive models. Association between JMJD1C SNP rs10822184 and (a) FSH, (b) Inhibin B, (c) Inhibin B/FSH ratio, (d) SHBG, (e) testosterone, (f) T x LH, (g) testis size by ultrasound, and (h) total sperm count. FSH and T x LH are presented on logarithmic scales; testosterone, SHBG, Inhibin B/FSH ratio, and total sperm count are presented on a cube root scale; and Inhibin B is presented on a square root scale in order to achieve normal distributions.

Supplementary Figure S3: Immunohistochemical staining of testis tissue from men with different rs7910927 genotypes. The antibody against JMJD1C revealed staining (brown colour) in the nucleus of Sertoli and Leydig cells and in the nucleus of germ cells until the round spermatid stage. The antibody directed against AR revealed staining in the nuclei of Sertoli cells, Leydig cells, and peritubular cells. No obvious differences were observed between staining patterns in biopsies with

different rs7910927 genotypes, albeit a somewhat stronger JMJD1C staining was observed among the TT biopsies. Scale bar depicts 100μm.

Supplementary Table S1 - *JMJD1C* SNP genotype frequencies in the young men

rs10822184 \ rs7910927	GG	TG	TT
CC	262	25	1
CT	1	468	19
TT	0	4	240

The two SNPs are highly linked. The Linkage Disequilibrium is: D’: 0.99 and R^2 = 0.90.

Supplementary Table S2 – rs7910927 in Fertile men

		GG	TG	TT	P-value
	N	Mean	Mean	Mean	
FSH (U/L)	310	3.21	3.15	2.72	0.150
Inhibin B (pg/mL)	310	193.35	198.26	196.74	0.867
InhibinB/FSH ratio	310	64.70	66.29	79.13	0.213
LH (U/L)	310	3.00	3.24	2.98	0.231
Testosterone (nmol/L)	308	17.96	17.96	16.53	0.372
Testosterone/LH ratio	308	6.07	5.64	5.63	0.492
Testosterone x LH	308	52.64	56.75	48.88	0.302
cFT (pmol/L)	308	376.85	381.10	355.73	0.559
cFT/LH ratio	308	139.59	130.67	130.01	0.606
Estradiol (pmol/L)	310	68.16	62.42	75.25	**0.036**
Testosterone/Estradiol ratio	308	304.12	334.24	276.91	0.064
cFT/Estradiol ratio	308	6.43	7.07	5.97	0.077
SHBG (nmol/L)	310	33.13	32.56	30.9	0.636
Volume (mL)	301	3.52	3.55	4.11	0.042
Concentration (millions)	301	74.94	58.55	59.64	0.135
Total spermcount (millions)	301	257.99	208.11	236.83	0.360
Morphologically normal sperm (%)	258	16.12	13.34	11.80	0.119
Motile sperm (AB) (%)	288	54.72	52.93	57.91	0.053
Testis size (orchidometer) (mL)	311	22.26	21.73	21.04	0.407
Testis size (ultrasound) (mL)	158	14.63	14.69	15.60	0.454

Associations between rs7910927 and male reproductive parameters in fertile men assuming additive genetic models. N: number of men. Values are geometric mean values. P-values below 0.05 are considered significant and are marked in bold.

Supplementary Table S3 - rs7910927 in Infertile men

		GG	TG	TT	P-value
	N	Mean	Mean	Mean	
FSH (U/L)	241	4.70	5.92	5.13	0.165
Inhibin B (pg/mL)	241	150.16	126.65	146.47	0.184
InhibinB/FSH ratio	241	39.03	26.92	33.67	0.166
LH (U/L)	241	3.97	4.04	3.80	0.730
Testosterone (nmol/L)	238	19.57	17.74	18.29	0.080
Testosterone /LH ratio	238	5.14	4.57	5.00	0.333
Testosterone x LH	238	75.79	70.08	67.99	0.394
cFT (pmol/L)	238	381.71	354.31	380.32	0.182
cFT/LH ratio	238	108.29	101.86	113.33	0.430
Estradiol (pmol/L)	241	68.34	64.83	68.09	0.510
Testosterone/Estradiol ratio	238	307.56	381.13	287.72	0.746
cFT/Estradiol ratio	238	5.98	6.73	5.87	0.813
SHBG (nmol/L)	242	37.43	35.31	33.48	0.202
Volume (mL)	239	3.67	3.57	3.97	0.318
Concentration (millions)	240	13.70	10.69	11.19	0.398
Total spermcount (millions)	239	50.78	37.43	46.81	0.309
Morphologically normal sperm (%)	219	3.00	3.86	2.27	0.108
Motile sperm (AB) (%)	215	33.92	28.75	31.07	0.229
Testis size (orchidometer) (mL)	246	18.38	17.48	19.08	0.085
Testis size (ultrasound) (mL)	243	12.25	12.00	12.84	0.433

Associations between rs7910927 and male reproductive parameters in infertile men assuming additive genetic models. N: number of men. Values are geometric mean values.

Supplementary Figure S1 - Flowchart over the exclusion criteria for the adult male cohorts

a

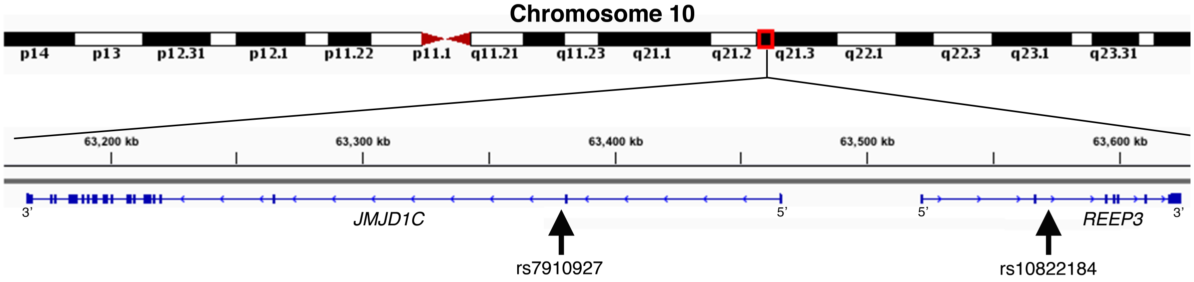

b

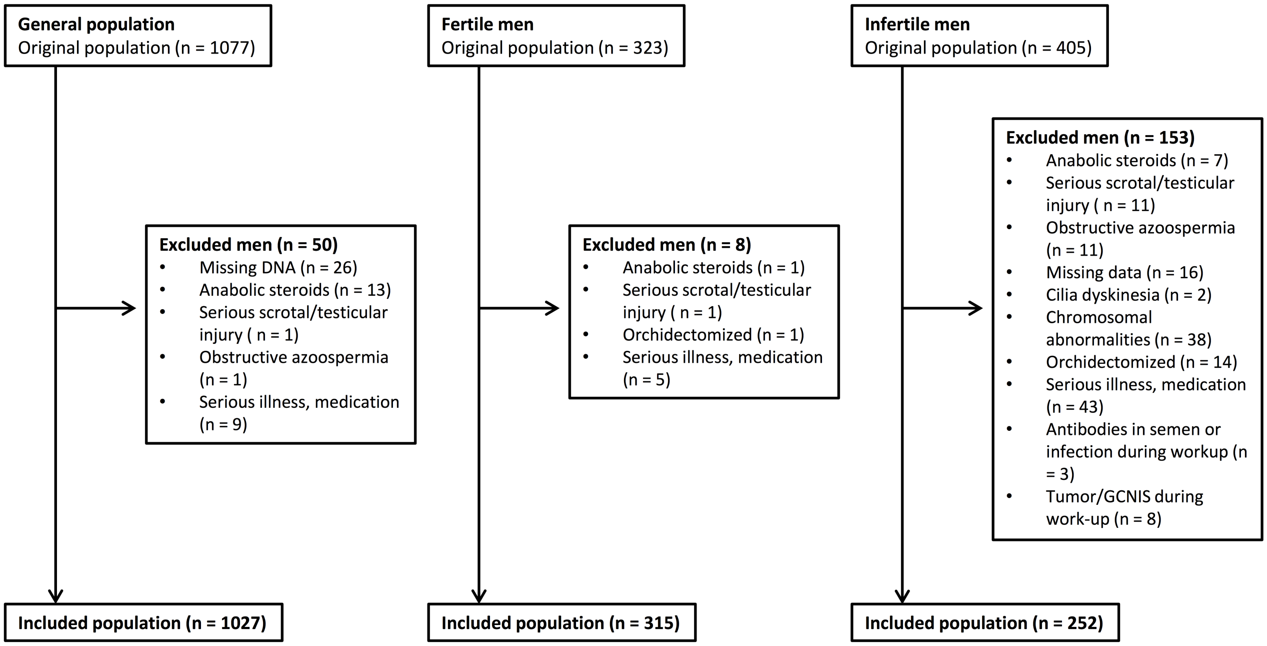

Supplementary Figure S2 - Boxplots of the association between reproductive parameters and rs10822184 in young men

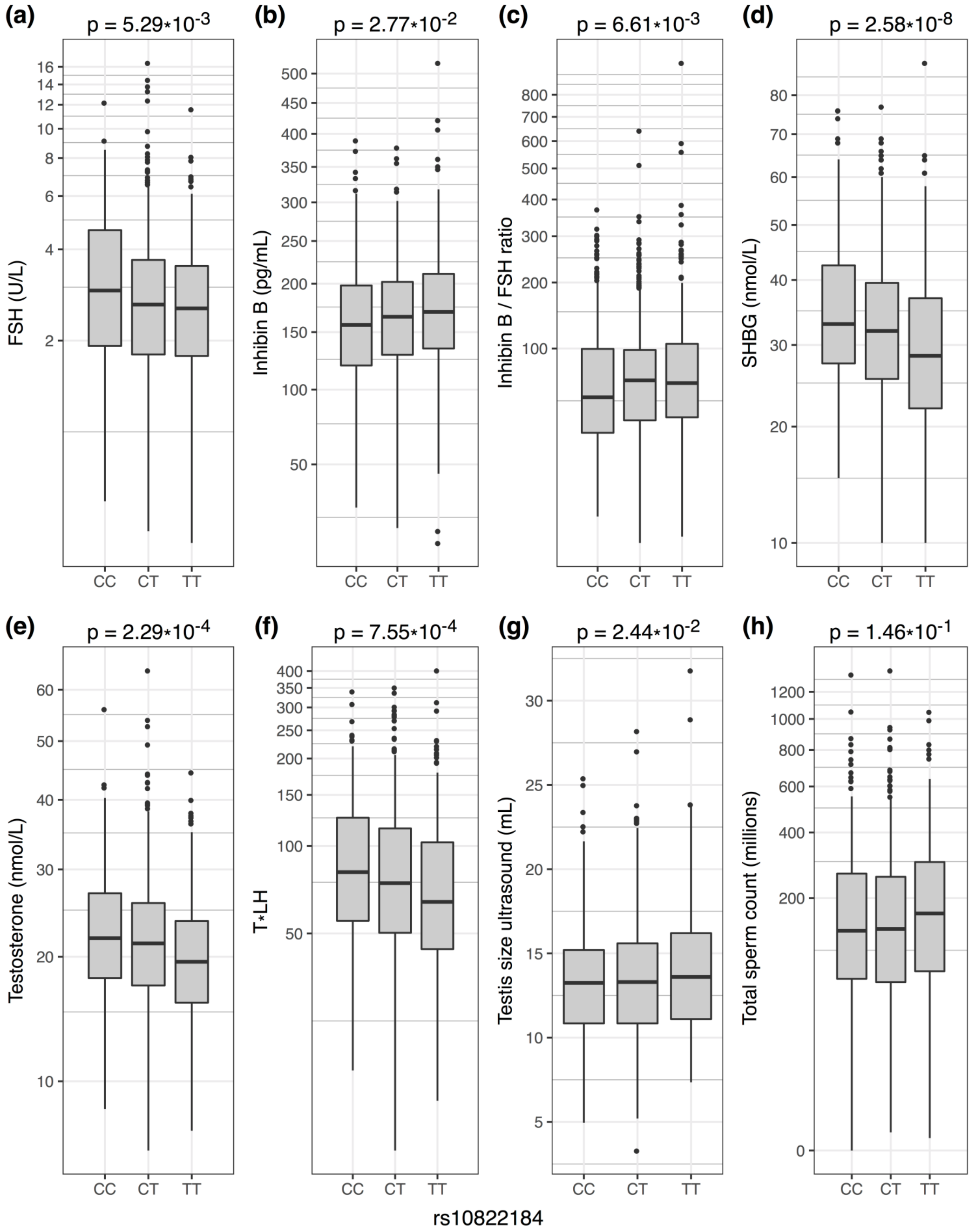
(a)
p = 5.29*10^-3
FSH (U/L)
(b)
p = 2.77*10^-2
Inhibin B (pg/mL)
(c)
p = 6.61*10^-3
Inhibin B / FSH ratio
(d)
p = 2.58*10^-8
SHBG (nmol/L)
(e)
p = 2.29*10^-4
Testosterone (nmol/L)
(f)
p = 7.55*10^-4
T*LH
(g)
p = 2.44*10^-2
Testis size ultrasound (mL)
(h)
p = 1.46*10^-1
Total sperm count (millions)
CC
CT
TT
rs10822184

Supplementary Figure S3 - Immunohistochemical staining of testis tissue from men with different rs7910927 genotypes.

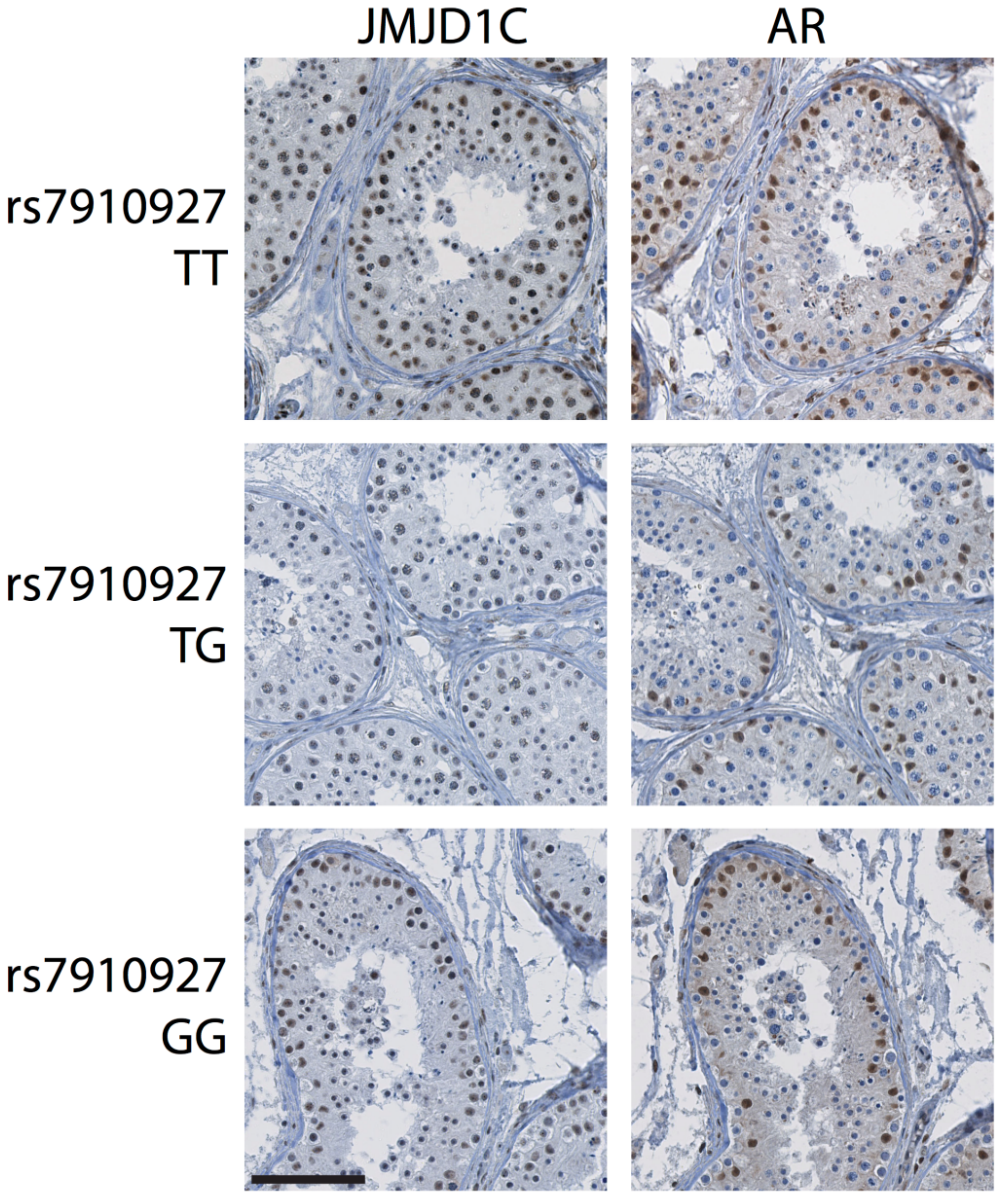

Supplement: Supplementary file 1 — Supplementary information [file 41598_2017_17575_MOESM1_ESM.pdf]
